# Influence of study characteristics, methodological rigour and publication bias on efficacy of pharmacotherapy in obsessive-compulsive disorder: a systematic review and meta-analysis of randomised, placebo-controlled trials

**DOI:** 10.1136/bmjment-2023-300951

**Published:** 2024-02-12

**Authors:** Sem E Cohen, Jasper Brian Zantvoord, Bram W C Storosum, Taina Kristiina Mattila, Joost Daams, Babet Wezenberg, Anthonius de Boer, Damiaan A J P Denys

**Affiliations:** 1 Psychiatry, Amsterdam UMC Locatie AMC, Amsterdam, The Netherlands; 2 Amsterdam Neuroscience Research Institute, Amsterdam, The Netherlands; 3 Medicines Evaluation Board, Utrecht, The Netherlands; 4 Medical Library, Amsterdam UMC Locatie AMC, Amsterdam, The Netherlands; 5 Division of Pharmacoepidemiology and Clinical Pharmacology, Utrecht University, Utrecht, The Netherlands

**Keywords:** Adult psychiatry, Anxiety disorders, Data Interpretation, Statistical

## Abstract

**Question:**

We examined the effect of study characteristics, risk of bias and publication bias on the efficacy of pharmacotherapy in randomised controlled trials (RCTs) for obsessive-compulsive disorder (OCD).

**Study selection and analysis:**

We conducted a systematic search of double-blinded, placebo-controlled, short-term RCTs with selective serotonergic reuptake inhibitors (SSRIs) or clomipramine. We performed a random-effect meta-analysis using change in the Yale-Brown Obsessive-Compulsive Scale (YBOCS) as the primary outcome. We performed meta-regression for risk of bias, intervention, sponsor status, number of trial arms, use of placebo run-in, dosing, publication year, age, severity, illness duration and gender distribution. Furthermore, we analysed publication bias using a Bayesian selection model.

**Findings:**

We screened 3729 articles and included 21 studies, with 4102 participants. Meta-analysis showed an effect size of −0.59 (Hedges’ G, 95% CI −0.73 to −0.46), equalling a 4.2-point reduction in the YBOCS compared with placebo. The most recent trial was performed in 2007 and most trials were at risk of bias. We found an indication for publication bias, and subsequent correction for this bias resulted in a depleted effect size. In our meta-regression, we found that high risk of bias was associated with a larger effect size. Clomipramine was more effective than SSRIs, even after correcting for risk of bias. After correction for multiple testing, other selected predictors were non-significant.

**Conclusions:**

Our findings reveal superiority of clomipramine over SSRIs, even after adjusting for risk of bias. Effect sizes may be attenuated when considering publication bias and methodological rigour, emphasising the importance of robust studies to guide clinical utility of OCD pharmacotherapy.

**PROSPERO registration number:**

CRD42023394924.

**WHAT IS ALREADY KNOWN ON THIS TOPIC**

- Selective serotonergic reuptake inhibitors and clomipramine are widely used in pharmacological treatment of obsessive-compulsive disorder.
- The National Institute for Health and Care Excellence guidelines and the American Psychiatric Association guidelines recommend using SSRI’s as a first step in the treatment of obsessive-compulsive disorders.
- In clinical trials, it is currently unclear if OCD symptom reduction is mediated by trial characteristics and by the quality of the trial.

**WHAT THIS STUDY ADDS**

- Most short-term efficacy trials of SSRI’s and clomipramine in OCD have at least some methodological flaws, and studies at high risk of bias are more effective.
- Clomipramine remains more efficacious than SSRI’s, even after correcting for risk of bias.
- Results show an indication for publication bias which inflates effect size estimation.

**HOW THIS STUDY MIGHT AFFECT RESEARCH, PRACTICE OR POLICY**

- As short-term efficacy of pharmacotherapy in OCD might be overestimated, there is a need for re-evaluation of current evidence, and for novel high-quality intervention trials.

## Background

Obsessive-compulsive disorder (OCD) is characterised by persistent thoughts, images and/or sensory perceptions that cause distress and repetitive behaviours performed in order to temporarily reduce distress. Its global lifetime prevalence is 2%.^1^ Clinical presentation is heterogeneous, but without treatment OCD may profoundly impair quality of life and social functioning. Selective serotonin reuptake inhibitors (SSRIs), cognitive–behavioural therapy with exposure and response prevention, or a combination of both is currently recommended for management of OCD.^2^ Clomipramine was the first effective pharmacotherapeutic intervention, but its side effect profile renders it a secondary option to SSRIs.^3^

In the context of major depressive disorder (MDD), concerns have been raised regarding overestimation of SSRI efficacy due to flaws in trial quality.^4–6^ For instance, a recent reanalysis of a meta-analysis of randomised SSRI trials in MDD showed all studies to be of high or unclear risk of bias. Moreover, another meta-analysis found a small, clinically irrelevant symptom reduction by SSRIs.^7 8^ However, these results are derived from average treatment effects and did not take modifiers of effect into account. Furthermore, a decreasing effect size might be the result of increasing placebo effect or increasing treatment resistance of the trial population.^9 10^ Whether overestimation of efficacy pervades in studies examining SSRIs in OCD as well remains currently unanswered.

Besides quality, study-level characteristics such as sponsor status, number of treatment arms and trial population have been found to influence trial success.^11 12^ For OCD, there is currently a lack of information on the influence of randomised controlled trial (RCT) methodology on efficacy, even though this information could contribute to optimising future trial design.

Recent evidence suggests that publication bias exaggerates pharmacotherapy efficacy in MDD, with more pronounced effect size inflation in older trials.^13^ For OCD, a study examining publication bias compared efficacy measures of published trials with original Food and Drug Administration (FDA) data. They found a pooled effect size of 0.39 Hedges’ G according to the US FDA, compared with 0.45 according to the published scientific literature.^14^ This increase was non-significant; however, this analysis only included SSRI trials submitted for FDA approval, potentially skewing the representation of reporting bias for OCD pharmacotherapy. Also, publication bias is often identified using funnel plot models that focus on small-study effects, while selection models that focus on biases in publishing of significant studies might be more sensitive to publication bias.^15^ ^16^ In addition, Bayesian approaches to selection models are especially suited for smaller meta-analyses.^17 18^

## Objective

In this study, we examined the effect of study-level characteristics on the efficacy of pharmacotherapy in placebo-controlled OCD trials. Furthermore, we analysed the influence of risk of bias on effect size and investigated publication bias employing a Bayesian approach.

We hypothesised an inverse correlation between risk of bias and effect size and that publication bias could exaggerate efficacy. We predicted larger effect sizes in industry-sponsored studies, older studies, studies with multiple treatment arms, with placebo run-ins and with fixed-dose regimens. We also hypothesised that studies with participants having shorter illness duration, higher baseline severity and younger age show higher efficacy.

## Study selection and analysis

### Search strategy

We searched Embase, Medline, PsycINFO and the Web of Science Conference Proceedings Index on 22 February 2023. Additionally, we searched the WHO International Clinical Trial Registry Platform search portal for registered studies, did a scoping search on Cochrane CENTRAL and used websites of several major conferences to search for unpublished literature or conference proceedings. We checked the included articles for references and conducted citation screening. For a detailed account of our search strategy, see online supplemental material.

10.1136/bmjment-2023-300951.supp1 Supplementary data

### Screening and inclusion

Two investigators (SEC and BW) independently screened studies using Rayyan.^19^ We included double-blind RCTs for monotherapy with an SSRI or clomipramine in adult patients (18 years and older) with OCD. We included a non-selective patient population suffering from OCD with all subtypes. We included short-term studies with a primary endpoint up to 16 weeks using the Yale-Brown Obsessive-Compulsive Scale (YBOCS).

If studies did not publish quantified data or if we were unable to retrieve the full text, we contacted the authors to request the information necessary for our analysis. If we were unable to retrieve this information, we excluded the study. Inclusion or exclusion conflicts were resolved by consensus, or if necessary through a consensus meeting with the coauthors. We prespecified the methods in the PROSPERO database for systematic reviews (registration number CRD42023394924).

### Data extraction

Two authors (SEC and BWCS) extracted the following data from the included studies: mean age and gender, number of participants in active or placebo group, exclusion criteria, intervention and dosing regimen, washout period, time to primary endpoint and difference in YBOCS response for placebo and intervention. If a study used a fixed-dosing regimen with multiple doses, we subdivided each dosing group and compared them with a proportionally reduced placebo group. Also, we extracted publication year, sponsor status and sponsor name, country or countries of trial site(s), use of a placebo run-in, and number of trial site(s). To assess risk of bias, we used the Cochrane Risk of Bias V.2.0 tool.^20^ Risk of bias, subdivided into low, some concerns and high risk of bias, was assessed by SEC and BWCS. Discrepancies were discussed in the entire research team in order to reach consensus.

### Meta-analytic method

As the primary outcome, we used the mean change in YBOCS at primary study endpoint compared with baseline. If mean change scores were not reported, we used difference in YBOCS at study endpoint after ensuring baseline symptom severity to be balanced across intervention arms. For effect size, we used Hedges’ G, with a standard CI of 95% using Knapp-Hartung adjustments.^21 22^ Assuming between-study variability, we used a random-effects model for pooling effect sizes, with a restricted maximum likelihood estimator to calculate the estimated SD of the true mean difference (τ).^23^ We chose to pool clomipramine and SSRIs into one group and carried out a meta-regression for potential moderating effect of intervention type (clomipramine or SSRI). We analysed SSRIs as one group as they share their primary mechanism of action even though they differ in chemical structure.^24 25^

We performed meta-regression for mean age, gender distribution, mean duration of illness, mean baseline severity, use of two or more trial arms, sponsor status, use of a placebo run-in phase, risk of bias (high risk yes/no) and publication year. Since we use a multitude of single regressions, we adjusted the significance threshold by adjusting halfway p=0.05 and the Bonferroni adjustment, that is, p=0.010. If moderators accounted for heterogeneity, we included them in a multiple meta-regression analysis. In order to avoid multicollinearity, we tested for prediction correlators. If highly correlated (r>0.8), we included the moderator causing the highest amount of heterogeneity. For sponsorship status, we distinguished between studies that were fully sponsored by pharmaceutical companies, publicly funded studies, privately funded studies by any other institution than a pharmaceutical company and studies in which only medication was reimbursed by a pharmaceutical company. Additionally, we performed a separate meta-analysis for the SSRI group only, using the same methodology.

Effect of study quality was evaluated in a separate analysis by excluding studies that were at high risk of bias. For the moderating effect of trial quality, we included a sensitivity analysis excluding the randomisation process from the risk of bias assessment, since randomisation processes are currently defined more strictly compared with when the original studies were published.^26^

### Publication bias

For publication bias analysis, we performed a fully Bayesian Copas analysis using set, weakly informative, priors. Thus, we were be able to quantify the magnitude of publication bias in our selected studies using a measure D, and calculate the difference between corrected and non-corrected effect sizes, using a Bayesian meta-analysis. Additionally, we performed a corrected Egger’s test and used funnel plot for visual inspection of publication bias.

For meta-analytic estimation of pooled effect sizes, as well as for meta-regression and conventional publication bias quantification, we used the metafor and dmetar packages in R.^27 28^ For Bayesian analysis for publication bias, we used the Robustbayesiancopas package in R.^18^

## Findings

### Search results and study description

**Figure 1:**
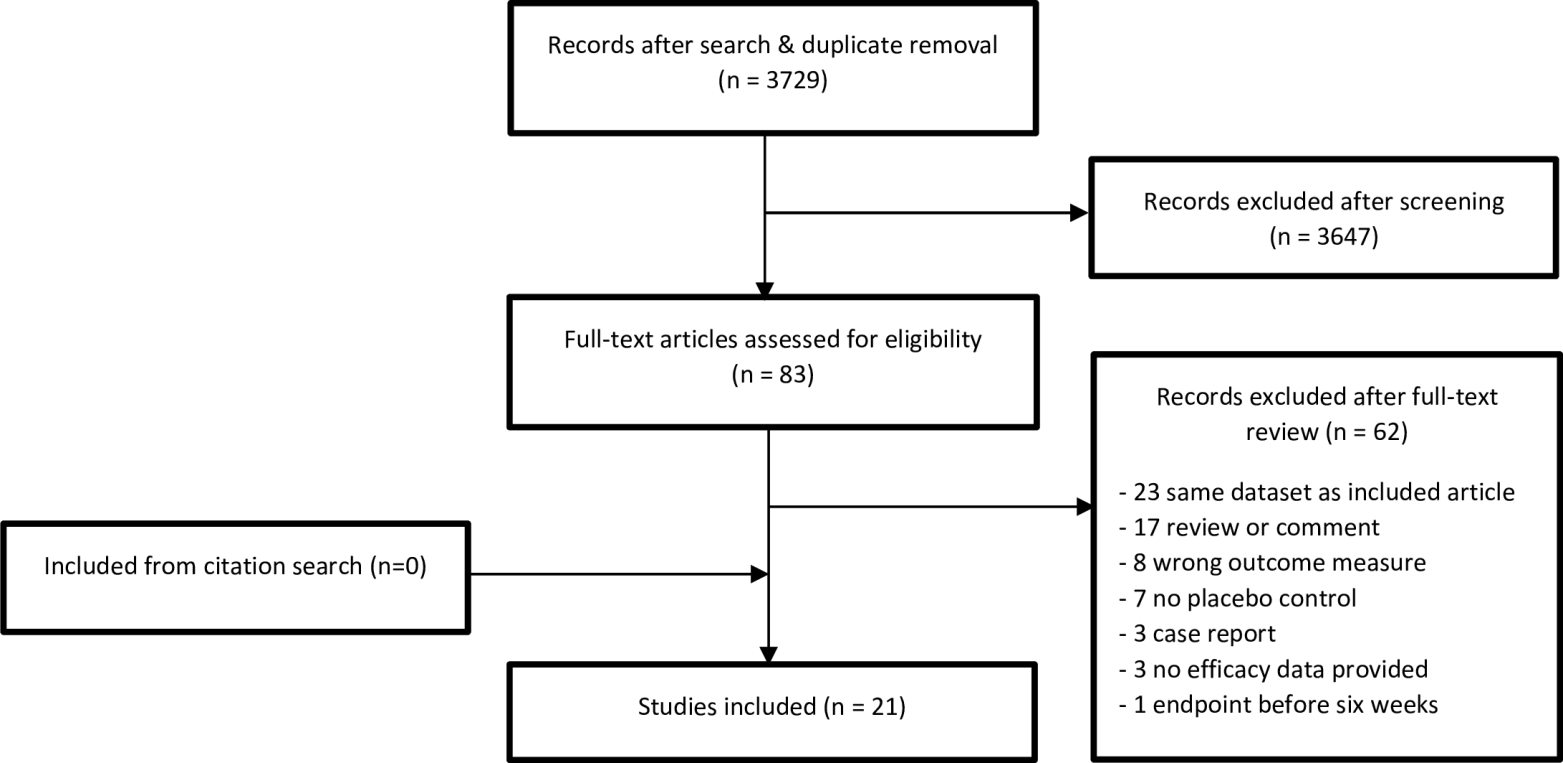
Flow chart of inclusion and exclusion of studies.

Our search yielded 3729 articles, of which we excluded 3646 after screening of titles and abstracts. Of the 83 articles we included for full-text screening, we included 20 papers, one of which reported on two separate studies, for a total of 21 studies.^29^ For a full account of our inclusion and exclusion, please refer to figure 1 and online supplemental material. The 21 included studies contained a total of 4102 participants, with 739 participants in the clomipramine RCTs and 3363 in the SSRI RCTs. 51 per cent of the participants were male. We included four studies focused exclusively on clomipramine, two of which were combined in a single manuscript. One three-armed study compared paroxetine with clomipramine and placebo. Of the included studies, all except four used a placebo run-in phase. Most studies were fully sponsored by a pharmaceutical company. Nine studies were supported by a grant independent from the producing company, most of which were granted free use of medication. The total number of included patients in independent studies was 232, against 3870 patients in sponsored studies. Nine studies used a fixed-dose regimen, one of which was a clomipramine study. All variables for meta-regression were available, except for mean duration of illness which was not mentioned in eight studies, and information on placebo run-in, which was unavailable in one article. For a full overview of study characteristics, see table 1 and online supplemental material.

**Table 1:** Study characteristics

Study	Drugs	Dose	Intervention/placebo, n (%)	Gender (M/F) (%)	Mean age (SD)	Baseline YBOCS (SD)	Treatment duration (weeks)	Sponsor	Duration of illness (years) (SD)	Primary outcome	Placebo run-in (weeks)
CSG 1, 1991^29^	Clomipramine	100–300, flexible dose	118/120 (50/50)	93/125 (43/57)	35.4 (10.6)	26.2 (5.5)	10	Ciba-Geigy	15 (10.3)	YBOCS week 10	2–4
CSG 2, 1992	Clomipramine	100–300, flexible dose	123/129 (49/51)	128/135 (49/51)	35.6 (10.6)	26.7 (4.9)	10	Ciba-Geigy	16.3 (10.7)	YBOCS week 10	2–4
Chouinard, 1990	Sertraline	50–200 mg, dose-finding	43/44 (49/51)	74/13 (85/15)	37 (11.8)	23 (5.5)	8	Pfizer	10 (12.0)	YBOCS week 8	1
Foa, 2005	Clomipramine	200–250 mg	36/26 (58/42)	34/28 (55/45)	34.8 (11.3)	23 (4.3)	12	NIMH	16 (10.7)	YBOCS week 12	0
Goodman, 1996	Fluvoxamine	100–300 mg	78/78 (50/50)	78/78 (50/50)	36.7	23.3 (6.0)	10	Solvay	16.5	YBOCS week 10	2–6
Goodman, 1989	Fluvoxamine	100–300 mg	21/21 (50/50)	19/23 (45/55)	37 (13)	25.3 (6.0)	6	NIMH, drugs by Solvay	15 (12)	YBOCS week 6	0
Greist, 1995	Sertraline	50, 100, 200 mg	79, 81, 80/84 (74/26)	191/133 (59/41)	38.7 (13)	23.7 (5.2)	12	Pfizer	5.3 (9)	YBOCS week 12	1
Hollander, 2003	Paroxetine	20, 40, 60 mg	88, 86, 85/89 (74/26)	256/92 (74/26)	41.3 (13.2)	25.5 (10.5)	12	SmithKline Beecham	–	YBOCS week 12	2
Hollander, 2003	Fluvoxamine	100–300 mg, flexible	127/126 (50/50)	92/161 (36/64)	37.4 (17.5)	26.4 (4.8)	12	Solvay	16.4 (19.1)	YBOCS week 12	1
Jenike, 1989	Clomipramine	200–300 mg, flexible	13/14 (48/52)	13/14 (48/52)	39.4 (10.5)	25.7 (5.0)	10	Ciba-Geigy	–	YBOCS week 10	2
Jenike, 1997	Fluoxetine	80 mg, fixed	23/21 (52/48)	23/21 (52/48)	35 (12.5)	19 (5.8)	10	NIMH grant, research fund	–	YBOCS week 10	2
Jenike, 1990	Fluvoxamine	50–300 mg, flexible	18/20 (47/53)	20/18 (47/53)	36 (11.1)	22.7 (4.8)	10	Partly by Solvay	18.5 (8.3)	YBOCS week 10	2
Kamijima, 2004	Paroxetine	40–60 mg, flexible	94/94 (50/50)	71/117 (38/62)	38 (12.1)	23.9 (4.6)	12	GSK	10.5 (9.5)	YBOCS week 12	1
Kronig, 1999	Sertraline	50–200 mg, flexible	86/81 (52/48)	92/75 (55/45)	36.5 (11.5)	25.2 (4.0)	12	Pfizer	17.0 (11.4)	YBOCS week 12	1
Mallya, 1992	Fluvoxamine	50–300, flexible	14/14 (50/50)	14/14 (50/50)	38	21.15 (5.7)	10	Not sponsored	–	YBOCS week 12	2
Montgomery, 2001	Citalopram	20, 40, 60 mg, fixed	102, 98, 100/101 (75/25)	184/217 (46/64)	37.8 (11.5)	25.6 (4.0)	12	Lundbeck	15.9 (11.6)	YBOCS week 12	1
Montgomery, 1993	Fluoxetine	20, 40, 60 mg, fixed	52, 52, 54/56 (74/26)	114/100 (53/47)	37 (12.0)	23.9 (6.2)	8	Lilly	–	YBOCS week 8	1
Nakatani, 2005	Fluvoxamine	100–200 mg, flexible	10/8 (56/44)	6/12 (33/77)	34.24 (7.0)	29.3	12	Government research grant	–	YBOCS week 12	–
Stein, 2007	Escitalopram, paroxetine	Escitalopram 10, 20 Paroxetine 40, fixed	113, 114/117/114 (49/26/25)	197/261 (43/57)	38 (11.8)	27.1 (4.0)	25	Lundbeck	4.1 (5.3)	YBOCS week 12	0
Tollefson, 1994	Fluoxetine	20, 40, 60 mg, fixed	87, 89, 90/89 (75/25)	159/196 (45/55)	37 (11.8)	22.7 (5.5)	12	Eli Lilly	–	YBOCS week 12	1
Zohar, 1996	Paroxetine, clomipramine	20–60 mg, 50–250 mg, flexible	201/99/99 (48/24/24)	209/190 (52/48)	38	26	12	SmithKline Beecham	15	YBOCS week 12	2

Concise overview of included placebo-controlled randomised clinical trials on OCD pharmacotherapy. Where reported in the original publication, we added SD to mean age, severity and duration of illness.

CSG, Clomipramine Study Group; F, female; GSK, GlaxoSmithKline; M, male; n, number; NIMH, National Institute of Mental Health; OCD, obsessive-compulsive disorder; YBOCS, Yale-Brown Obsessive-Compulsive Scale.

### Risk of bias

**Figure 2:**
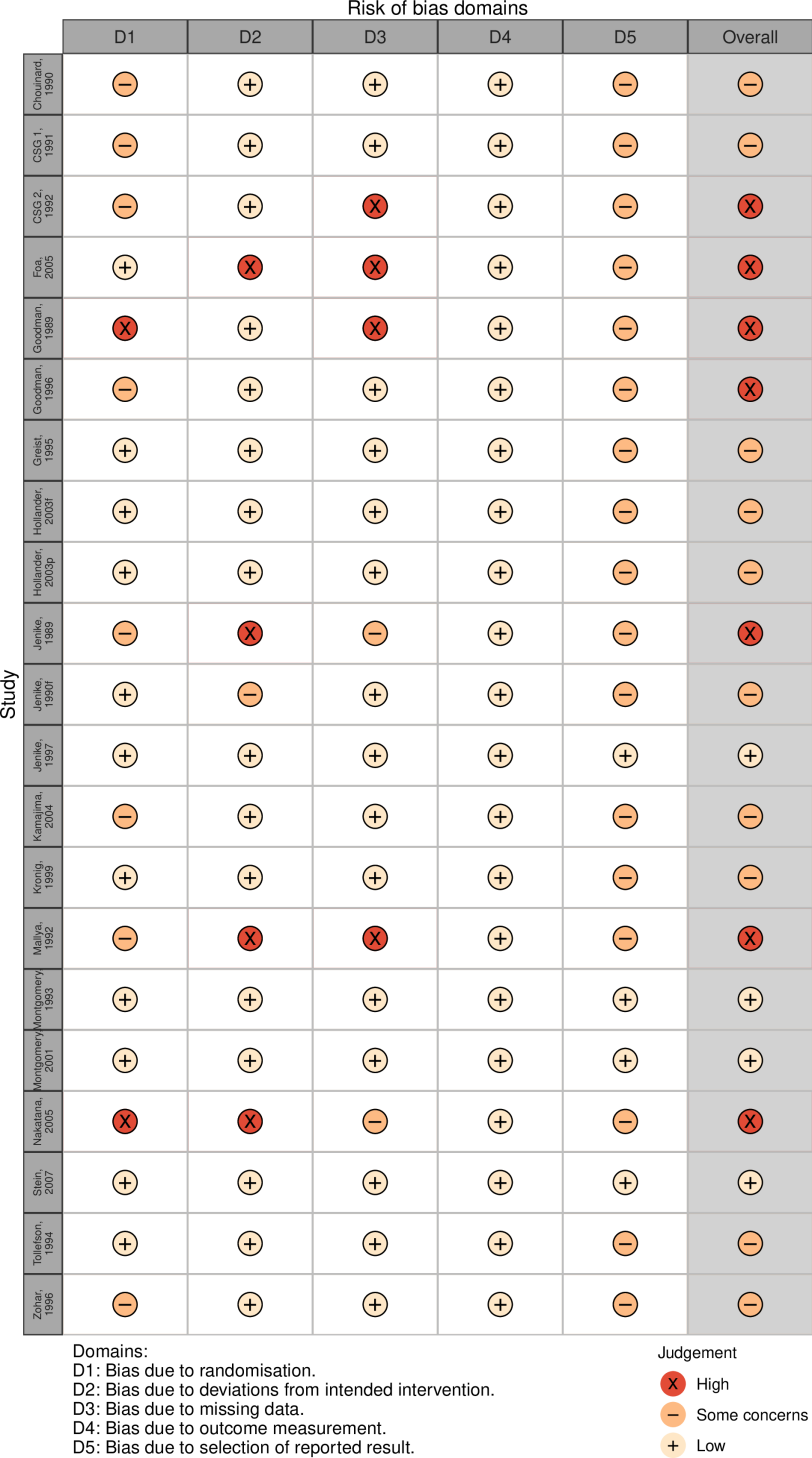
Risk of bias assessment for all studies using the Cochrane Risk of Bias V.2.0 tool. CSG, Clomipramine Study Group.

In total, 4 out of 21 included studies were judged as having a low risk of bias (see figure 2). Seven studies were at high risk of bias and 10 had some concerns. Concerns on randomisation resulted mostly from absence of reporting on the allocation sequence generation process and allocation concealment. Regarding assignment of intervention, four studies were at high risk of bias for not performing an intent-to-treat analysis, with dropouts possibly leading to attrition bias. Four studies excluded more than 10% of patients from the efficacy analysis. Risk of bias in measurement of outcome was low in all studies. In reporting of results, all but three studies did not report on the use of a prespecified analysis plan. For a full account of the risk of bias assessment, see online supplemental material. Our predefined sensitivity analysis excluding the randomisation process from the risk of bias assessment did not change overall bias results.

### General meta-analysis

**Figure 3:**
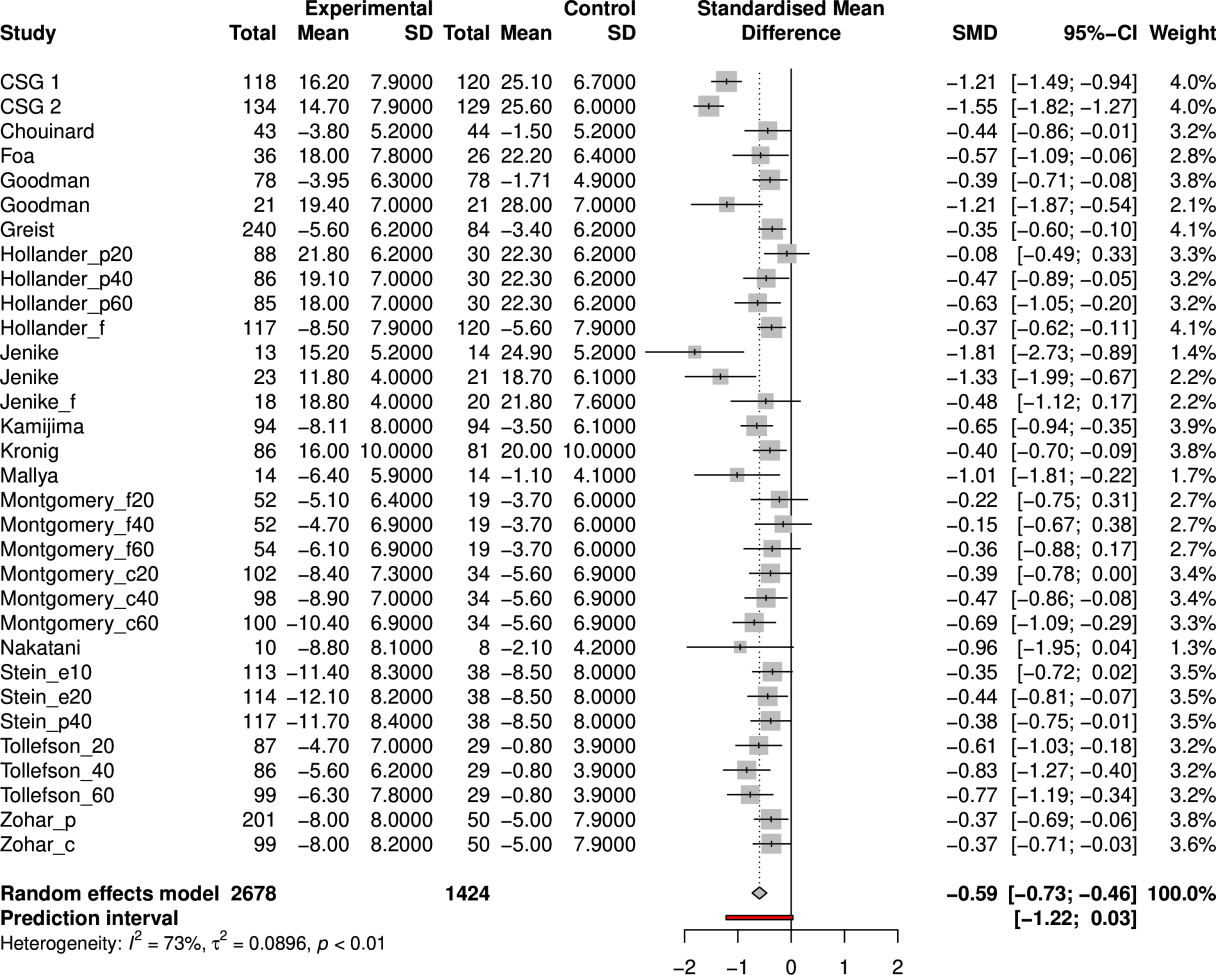
Forest plot of random-effects meta-analysis of included studies. c, clomipramine; CSG, Clomipramine Study Group; e, escitalopram; f, fluvoxamine; p, paroxetine; SMD, standardised mean difference.

A random-effects meta-analysis with Knapp-Hartung adjustments resulted in a standardised mean difference (SMD) of −0.59 (Hedges’ G, 95% CI −0.73 to −0.46; see figure 3), which is equal to a mean difference of 4.2 points on the YBOCS. The test for heterogeneity demonstrated a significant level of variability across the samples (Q=114, p<0.0001), with an estimated tau of 0.30, suggesting considerable between-study variance and an I^2^ value of 73%, indicating a high proportion of the observed variance reflects real differences in effect sizes. Heterogeneity was further demonstrated by the 95% prediction interval, which ranged from −1.22 to 0.03. Random-effects meta-analysis for SSRIs specifically resulted in an effect size of −0.47 SMD (Hedges’ G, 95% CI −0.56 to −0.39) with low heterogeneity across studies (I^2^=16.0%, tau<0.0001) (see online supplemental figure S2).

### Meta-regression

After controlling for baseline differences, studies using clomipramine had a higher efficacy than SSRI studies (β=−0.55, 95% CI −0.24 to −0.9686, p=0.0011), as were studies at high risk of bias (β=−0.51, 95% CI −0.19 to −0.82, p=0.0029). Publication year was negatively associated with YBOCS change (suggesting that RCT efficacy decreases over time; β=0.028, 95% CI 0.0053 to 0.050, p=0.017). Furthermore, studies that were fully sponsored by a pharmaceutical company had a smaller effect size than non-sponsored or partially sponsored studies (β=0.46, 95% CI 0.092 to 083, p=0.016). Studies using two intervention arms had a higher efficacy compared with studies using one intervention arm (β=−0.33, 95% CI −0.061 to 0.60, p=0.016). Using our corrected significance threshold (p=0.010), publication year, sponsor status and number of study arms did not reach significance level. We performed a separate meta-regression in which dosing arms of fixed-dose studies were combined in one intervention arm, which led to a non-significant influence of the number of study arms on efficacy (see online supplemental material). No other regression results differed in these sensitivity analyses. Regarding baseline characteristics, increased mean age at baseline was associated with a decrease in efficacy (β=0.075, 95% CI 0.0074 to 0.14, p=0.031), which was insignificant after correcting for multiple analyses. Use of a placebo run-in, use of fixed or flexible dose, illness duration at baseline, baseline YBOCS and gender were not associated with changes in efficacy (see table 2 for the results of single meta-regression models).

**Table 2:** Regression coefficients of single regressions

Predictor	Beta coefficient	95% CI lower	95% CI upper	P value
Categorical predictors				
High risk of bias	−0.51	−0.82	−0.19	0.0029
Clomipramine use	−0.55	−0.86	−0.23	0.0011
Fully sponsored	0.46	0.1092	0.83	0.016
Two-armed intervention trial	−0.32	−0.59	−0.063	0.017
Use of placebo run-in	−0.029	−0.40	0.34	0.88
Flexible dose	−0.26	−0.52	0.0078	0.057
Continuous predictors				
Publication year	0.028	0.0053	0.050	0.017
Mean age	0.075	0.0074	0.14	0.031
Mean severity	0.016	−0.062	0.095	0.68
Duration of illness	−0.020	−0.055	0.016	0.26
Percentage of male	0.0078	−0.0066	0.022	0.28

Subsequently, we performed a multiple meta-regression analysis for significant predictors. Testing for multicollinearity showed no redundant variables (see online supplemental table S2 for the multicollinearity table). We included risk of bias, clomipramine, sponsor status, number of intervention arms, mean age and publication year in our multiple meta-regression model using a mixed effect of maximum likelihood. Using our best-performing meta-regression model, we found that when correcting for high risk of bias clomipramine remained significantly correlated with a higher effect size compared with SSRI (β=−0.43, 95% CI −0.74 to −0.12, p=0.0085; table 3). For additional information regarding model performance and selection, see online supplemental material.

**Table 3:** Regression coefficients of multilevel meta-regression for the model, including studies with high risk of bias and clomipramine use

Predictor	Beta coefficient	95% CI lower	95% CI upper	P value
High risk of bias	−0.33	−0.64	−0.0085	0.044
Clomipramine use	−0.43	−0.74	−0.12	0.0085

### Publication bias

**Figure 4:**
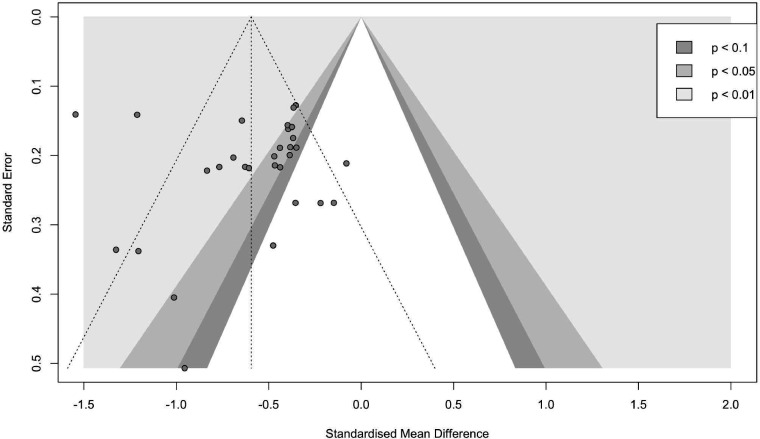
Funnel plot for visual inspection of publication bias.

For the full sample, visual inspection of the funnel plot (figure 4), as well as the Egger’s linear regression test of funnel plot asymmetry, as implemented using the method by Pustejovsky, shows no indication of publication bias (t=0.23, p=0.82). In contrast, using a Bayesian Copas selection model, a moderate amount of publication bias was found (D=0.48). After adjusting for publication bias, efficacy was reduced with an SMD of 0.11, from −0.53 (95% credible interval −0.64 to −0.42) to −0.42 (95% credible interval −0.60 to −0.22) using Bayesian analysis for determining efficacy.

### GRADE assessment

We used the Grading Recommendations Assessment Development Evaluation (GRADE) to evaluate the certainty of evidence. 30 Due to risk of publication bias and the large percentage of studies at high risk of bias or with some concerns, we graded the certainty of evidence as low, meaning that confidence in the effect estimate is limited and the true effect may substantially differ from the estimated effect (online supplemental table S7).^30^

## Conclusions and clinical implications

In our meta-analysis of RCTs, we found that pharmacotherapy for OCD has a medium effect size in favour of intervention compared with placebo, amounting to 4.2 points on the YBOCS. The results were heterogeneous, with studies at high risk of bias being more likely to lead to a larger effect than studies that were at low risk of bias or had some concerns. Clomipramine was more efficacious than SSRIs, even after correcting for studies at high risk of bias. Furthermore, using a Bayesian analysis, we found a moderate risk of publication bias, with a small decrease in estimated efficacy after correcting for publication bias.

Our efficacy findings were comparable with those of an earlier meta-analysis and a recent network meta-analysis.^31 32^ The advantage of clomipramine over SSRIs is also in line with earlier evidence.^33^ However, in contrast to our analysis, Skapinakis *et al* 31 found that clomipramine was not significantly more efficacious than SSRIs and that any non-significant differences further dissipated when considering studies with low risk of bias. This result might be explained by the fact that the authors conducted a network analysis, also including head-to-head clinical trials. Furthermore, including only studies with low risk of bias is based on the stern assumption that studies with some methodological shortcomings do not have any added empirical value and it depletes power for subgroup analysis. Alternative explanations for clomipramine’s higher efficacy include lower quality and older studies conducted before SSRIs, suggesting the population was medication-naïve with fewer non-responders.^33 34^ Although studies did not register the degree of earlier non-response, clomipramine remained a significant predictor of treatment success after correcting for publication year.

After correction for multiple tests, we did not find an effect of participant characteristics on trial efficacy, in line with earlier literature.^35^ Furthermore, study characteristics such as publication year and amount of intervention arms were not significant after adjusting for multiple testing. We expected that pharmaceutical-sponsored studies would demonstrate higher efficacy than public or non-industry-funded studies, as found in a recent Cochrane review.^36^ However, we saw a negative influence of industry sponsorship, even though this effect was not significant after correcting for multiple tests. This contrasts the argument of investigator bias inflating effect size. We hypothesise that this rather reflects the paucity of non-sponsored studies as besides being older they are relatively small, single-centre and therefore more likely to be homogeneous and non-generalisable. Although a placebo-run in phase has been found to increase efficacy by decreasing placebo response in depression trials, we were unable to demonstrate an effect of placebo run-in, possibly as almost all studies actually used a run-in phase.^37^ Notably, the European Medicines Agency guideline on clinical investigation of medicinal products for the treatment of OCD recommends against using a run-in phase as it might impair generalisation of the study results.^38^

Our meta-analysis is the first study on OCD pharmacotherapy to demonstrate an effect of publication bias on efficacy, using a Copas selection model with a Bayesian approach. Selection models, which assume that publication bias arises from selective publishing of statistically significant studies, are thought to be preferable over funnel plot methods for detection of publication bias.^15 39 40^ Moreover, Bayesian selection models circumvent the assumption of a normal distribution of effect sizes and is thought to enhance sensitivity in smaller meta-analyses.^18^ In the case of OCD, we conclude that there is clear evidence of effect size inflation due to publication bias in the literature and treatment benefit is diminished somewhat after correction.

We found only four studies with a low risk of bias and seven studies with a high risk. The remainder of the studies were deemed at some concern for risk of bias. Most studies did not have a predefined analysis plan or protocol, and most studies did not disclose the randomisation process or allocation procedure. Both might be explained by shifting publication and quality standards for RCTs, more specifically because almost all included studies were published before the 2004 requirement by journals for prospective registration in a public trial registry and before the FDA mandated preregistration in 2007.^41 42^ In fact, the most recent article we were able to include was published in 2007. In addition to critically appraising current evidence, we took into account the influence of risk of bias on effect size in OCD pharmacotherapy. To our knowledge, this is the first meta-analysis to do so and we found that studies at high risk of bias are correlated with an increased effect size. This finding raises concerns regarding effect size inflation in pivotal OCD pharmacotherapy trials and emphasises the need for (novel) high-quality evidence on SSRI and clomipramine efficacy in OCD.

### Limitations

Our meta-analysis does have some limitations. Although we did perform meta-regression for participant-level characteristics such as age, gender and illness severity, using aggregate data meta-analysis for these interactions is problematic, since results might be falsely positive due to ecological fallacy, in which treatment effects are confounded by a third, unknown factor.^43^ On the other hand, potential modifiers on the participant level might also be missed if studies did not show variation in aggregate measures. Specifically for inspection of patient-level modifiers, the designated research method would be an individual participant data meta-analysis (IPDMA), which to the best of our knowledge has not been performed yet, specifically for OCD pharmacotherapy.^44^ Furthermore, for the meta-regression, we relied on how the original manuscripts defined variables, which might vary significantly. Additionally, although we did search grey literature and found evidence for publication bias, we did not find any unpublished studies. This might be due to selective reporting by sponsors, where negative findings not only remain unpublished in full, but are not reported on any public domain. Finally, as we only included placebo-controlled trials, our results pertain to a clinical trial population which might decrease representativeness of real-world population, for instance, regarding comorbidity and treatment resistance.^45^

### Clinical implications and recommendations for future research

Although our findings extend current literature by supporting evidence on the efficacy of OCD pharmacotherapy, the effect size is diminished after correcting for methodological issues and publication bias.^33 46^ Furthermore, the fact that most placebo-controlled trials for OCD, based on registered medications, fall short on contemporary quality standards is a concerning matter.

When interpreting these results, we should also differentiate between statistical and clinical relevance. It is questionable whether a mean active medication versus placebo separation of 4.2 points on the YBOCS and less for SSRIs amounts to a noticeable change in illness severity. This is unknown as no generally accepted ‘minimal important difference’ exists for OCD, describing the smallest clinically noticeable decrease in symptoms. However, these are group-level effects, and within-group effects may vary significantly.

The advantage in the efficacy of clomipramine over SSRIs persisted even after correcting for risk of bias. However, direct head-to-head trials have not shown a clear increase in the efficacy of clomipramine over SSRIs, and clomipramine is known to have a more severe side effect profile compared with SSRIs, especially anticholinergic, cardiac and metabolic side effects.^34^ As such, we are not able to recommend concrete changes in clinical practice based on this article. Yet it is noteworthy that clomipramine, the inaugural approved pharmacological treatment for OCD in 1989, may remain one of our most potent treatment options.

Given that study effects are modest, susceptible to publication bias and influenced by the risk of bias, a new RCT reassessing the efficacy of OCD pharmacotherapy would hold significant clinical and regulatory importance. This trial should be conducted in a representative population, adhere to the latest quality standards and prioritise social functioning and quality of life, alongside symptom reduction. As the included RCTs focused on general efficacy rather than on predictors of treatment success, future research on the effect of symptom dimensions, the presence of sensory phenomena or metacognition on pharmacological treatment outcome would be valuable. However, we acknowledge the financial burdens associated with conducting such an RCT and the ethical concerns surrounding the inclusion of a placebo arm, thereby potentially depriving participants of an established first-line treatment for OCD.^20^ In light of these considerations, undertaking an individual IPDMA of OCD RCTs to first assess patient-level characteristics predicting improved treatment response would be both economically and ethically more favourable. Such subgroup analyses could refine interventions, identifying specific patient populations with enhanced treatment success likelihoods. Consequently, insights from an IPDMA could inform targeted cohorts for upcoming drug trials.

In summary, our findings suggest a pronounced efficacy for clomipramine over SSRIs, even after adjusting for risk of bias. While pharmacotherapy remains a viable therapeutic option for OCD, it is essential to recognise that reported effect sizes may be attenuated when considering publication bias and stringent methodological standards.

## Data Availability

All data relevant to the study are included in the article or uploaded as supplementary information. All data used in the meta-analysis are available in the manuscript and supplement. Code is available upon reasonable request.
