# Supplementary material for: Influence of study characteristics, methodological rigour and publication bias on efficacy of pharmacotherapy in obsessive-compulsive disorder: a systematic review and meta-analysis of randomised, placebo-controlled trials
Source: BMJ Ment Health. 2024 Feb 12;27(1):e300951. doi: 10.1136/bmjment-2023-300951 (PMC10862307; doi:10.1136/bmjment-2023-300951)
Supplement: Supplementary data [file bmjment-2023-300951supp001.pdf]

Supplementary material

Search strategy

Using a sensitivity-maximizing search, we included terms regarding the population and intervention, using an RCT filter. We did not use language or date restrictions. The search was conducted by author JD, clinical librarian and search specialist, in order to ensure a high degree of thoroughness. For population /domain being studied, we included the term obsessive-compulsive disorder and known synonyms. For intervention we used pharmacotherapy for OCD as recommended in the NICE treatment guideline and in the Anxiety and Depression Association of America treatment guideline. We searched for clomipramine, sertraline, paroxetine, fluoxetine and fluvoxamine, incuding known synonyms. Citalopram, escitalopram, mirtazapine and venlafaxine are not registered but are mentioned as treatment options in abovementioned guidelines, so we included them in our search.

We systematically searched Embase, Medline and PsycINFO. For the Embase search strategy, see figure **S1**. Comparable searches were done for Medline and PsycINFO**.** Additionally, we performed a scoping search of Cochrane CENTRAL which did not yield additional articles. We searched the WHI International Clinical Trial Registry Platform, as well as EUdraCT and clinicaltrials.gov. Additionally, we searched several symposia (ACNP, ECNP, Molecular Psychiatry, ADAA, IOCDF) for the last five years in order to included information that has not yet been published.

Figure S1: Embase search strategy

Ovid Embase Classic+Embase <1947 to 2023 February 21>
Search date: 22 February 2023
RCT filter: (Box 3.e Cochrane Highly Sensitive Search Strategy for identifying controlled trials in Embase: (2018 revision); Ovid format (Glanville et al 2019b: https://training.cochrane.org/handbook/version-6/chapter-4-tech-suppl))

#	Searches	Results
1	obsessive compulsive disorder/	30691
2	(obsessive compulsive disorder? or obsessive neurosis or compuls* neurosis or obsessive syndrome or ocd or Obsessive-Compulsive Scale or yale brown or ybocs or "y-bocs").ab,kw,ti.	27736
3	1 or 2 [obsessive compulsive disorder]	40445
4	(serotonin re?uptake inhibitor? or serotonin specific re?uptake inhibitor? or ssri? or 5?ht uptake inhibitor? or "5 Hydroxytryptamine uptake inhibitor?" or 5?ht re?uptake inhibitor? or "5 Hydroxytryptamine re?uptake inhibitor?").ab,kw,ti.	29794
5	(Sertralina or Sertraline or Sertralinum).ab,kw,ti,tn.	8132
6	(adjuvin or altruline or aremis or atruline or besitran or dominum or doxime or fatral or fridep or gladem or lesefer or lustral or nudep or seltra or serad or sercerin or serlain or serlift or sertralin or sertraline hydrochloride or sertranex or sertranquil or sosser or tatig or tresleen or zolof or zoloft or zosert).ab,kw,ti,tn.	3043
7	(arketis or aropax or aroxat or brisdelle or daparox or deroxat or dexorat or divarius or dropax or dropaxin or euplix or eutimil or frosinor or mesafem or motivan or optipar or paluxetil or paluxon or paroc or parogen or paroxedura or paroxet or paroxetin or paroxetina or paroxetine hydrochloride or paroxetine mesilate or paroxetine mesylate or paroxia or paxan or paxil or paxtine or paxxet or pexeva or serestill or sereupin or seroxat or setine or solben or syntopar or tagonis).ab,kw,ti,tn.	3219
8	(actan or adofen or afeksin or alzac or andep or andepin or ansilan or auroken or auscap or bioxetin or captaton or daforin or dagrilan or depren or deprex or deprexetin or deprexin or deprizac or deproxin or diesan or digassim or elizac or exostrept or felicium or fldiss or flotinal or floxet or fluctin or fluctine or fludac or flufran or fluketin or flunil or flunirin or fluohexal or fluoksetin or fluoksetyna or fluox or fluox-puren or fluoxac or fluoxeren or fluoxetin or fluoxetina or fluoxetine hydrochloride or fluoxifar or fluoxil or fluoxone or fluoxone divule or fluoxtab or fluronin or flusac or flustad or flutin or flutine or flux or fluxemed or fluxen or fluxet or fluxetil or fluxetin or fluxil or fluxomed or fluzac or fokeston or fontex or foxetin or foxtin or fropine or fuloren or gerozac or ladose or lanclic or lorien or lovan or luramon or magrilan or margrilan or meropan or modipran or mutan or nopres or nuzak or olena or oxactin or oxedep or plazeron or plinzene or pragmaten or prizma or proctin or prodep or prosac or prozac or prozamel or prozamin or prozep or prozit or psipax or qualisac or rapiflux or reconcile or reneuron or rowexetina or salipax or sanzur or sarafem or sartuzin or selfemra or serelsa or seromex or seronil or sinzacor sofelin or "stephadilat-s" or xeredien or zactin or zepax or zinovat).ab,kw,ti,tn.	129651
9	(Fluvoxamina or Fluvoxamine or Fluvoxaminum or luvox).ab,kw,ti,tn.	4710
10	(Citalopram or Citalopramum or Nitalapram or ctp).ab,kw,ti,tn.	18898
11	(Citalopram or Escitalopram or Escitalopramum).ab,kw,ti,tn.	12264
12	(Azamianserin or Mepirzapine or Mirtazapin or Mirtazapina or Mirtazapine or Mirtazapinum).ab,kw,ti,tn.	4052
13	(Venlafaxina or Venlafaxine or Venlafaxinum).ab,kw,ti,tn.	7243
14	("79617-96-2" or "61869-08-7" or "54910-89-3" or "54739-18-3" or "59729-33-8" or "128196-01-0" or "93413-69-5" or "303-49-1").ab,kw,rn.	107220
15	or/4-14 [SSRI's]	261967
16	(Chloroimipramine or Chlorimipramine or Clomipramina or Clomipramine or Clomipraminum or Monochlorimipramine).ab,kw,ti,tn.	4933
17	85650-52-8.ab,kw,rn.	1301
18	16 or 17 [clomipramine]	6219
19	Randomized controlled trial/	774446
20	Controlled clinical study/	469013
21	random$.ti,ab.	1945804
22	randomization/	98516
23	intermethod comparison/	294523
24	placebo.ti,ab.	366341
25	(compare or compared or comparison).ti.	627631
26	((evaluated or evaluate or evaluating or assessed or assess) and (compare or compared or comparing or comparison)).ab.	2722771
27	(open adj label).ti,ab.	106794
28	((double or single or doubly or singly) adj (blind or blinded or blindly)).ti,ab.	276766
29	double blind procedure/	210213
30	parallel group$1.ti,ab.	31735
31	(crossover or cross over).ti,ab.	124308
32	((assign$ or match or matched or allocation) adj5 (alternate or group$1 or intervention$1 or patient$1 or subject$1 or participant$1)).ti,ab.	410629
33	(assigned or allocated).ti,ab.	483810
34	(controlled adj7 (study or design or trial)).ti,ab.	446692
35	(volunteer or volunteers).ti,ab.	285865
36	human experiment/	639759
37	trial.ti.	403012
38	or/19-37 [RCT filter]	6286852
39	(error or erratum or letter or editorial).ab,kw,pt,ti.	2745590
40	3 and (15 or 18) and 38	2008
41	3 and (15 or 18) and 39	726
42	JPRN-UMIN000001726.cn.	0
43	or/40-42	2682

In- and exclusion of studies.

With full-text screening, we excluded 23 studies for using the same data from a trial that had already been presented in an earlier paper. We excluded eight studies for not using the YBOCS scale as outcome measure and seven for not using a placebo control group. 17 were excluded because they were a review or comment and three for presenting a case report. Three papers did not provide enough efficacy data to include them in our review, even after requests for information. We excluded one study for using 24 hours as endpoint, after administering intravenous clomipramine.

Risk of bias assessment.

We used the https://methods.cochrane.org/risk-bias-2, and through the official guidance document we filled in the risk of bias template for each study. See **table S1** , in which we simplified and summarized our risk of bias assessment.

Table S1: Risk of bias assessment

**Study**	**Domain 1**<br>**Randomization**	**Domain 2a**<br>**Assignment**	**Domain 3**<br>**Missing outcome data**	**Domain 4**<br>**Outcome measurement**	**Domain 5**<br>**Reporting**	**Overall risk of bias**
Chouinard, 1990, (1)	Some concerns<br>1.1./1.2 : Unclear allocation sequence / concealment	Low	Low	Low	Some concerns<br>5.1 No pre-specified analysis plan.	Some concerns
CSG 1, 1991, (2)	Some concerns<br>1.1./1.2 : Unclear allocation sequence / concealment	Low	Low	Low	Some concerns<br>5.1 No pre-specified analysis plan.	Some concerns
CSG 2, 1992, (2)	Some concerns<br>1.1./1.2 : Unclear allocation sequence / concealment	Low	3.1 not all ptcpts that were randomized, were analyzed	Low	Some concerns<br>5.1 No pre-specified analysis plan.	High
Foa, 2005 (3)	Low	High	High	Low	Some concerns<br>5.1 no pre-specified analysis plan	High
Goodman, 1989, (4)	Some concerns<br>1.1./1.2 : Unclear allocation sequence / concealment	Low	High<br>3.1 No ITT analysis, >10 % dropout	Low	Some concerns<br>5.1 No pre-specified analysis plan.	High
Goodman, 1996 (5)	High. 1.1/1.2 unclear, and<br>1.3 Allocation, age and gender all identical. Exceeds chance expecation	Low	Low	Low	Some concerns<br>5.1 No pre-specified analysis plan.	High

Greist, 1995 (6)	Low	Low	Low	Low	Some concerns<br><br>5.1 No pre-specified analysis plan.	Some concerns
Hollander, 2003f (7)	Low	Low	Low	Low	Some concerns<br><br>5.1 No pre-specified analysis plan.	Some concerns
Hollander, 2003p (8)	Low	Low	Low	Low	Some concerns<br><br>5.1 No pre-specified analysis plan.	Some concerns
Jenike, 1989 (9)	Some concerns<br><br>1.1./1.2 : Unclear allocation sequence / concealment	High<br><br>2.6/2.7 ITT unclear possible impact on results	Some concerns<br><br>3.1 ITT unclear, >10% dropout	Low	Some concerns<br><br>5.1 No pre-specified analysis plan.	High
Jenike, 1990f (10)	Low	Some concerns<br><br>2.6 no ITT, 2 dropouts, minor	Low	Low	Some concerns<br><br>5.1 No pre-specified analysis plan.	Some concerns
Jenike, 1997 (11)	Low	Low	Low	Low	Low	Low
Kamajima, 2004 (12)	Some concerns<br><br>1.1./1.2 : Unclear allocation sequence / concealment	Low	Low	Low	Some concerns<br><br>5.1 No pre-specified analysis plan.	Some concerns
Kronig, 1999 (13)	Low	Low	Low	Low	Some concerns<br><br>5.1 No pre-specified analysis plan.	Some concerns
Mallya, 1992 (14)	Some concerns<br><br>1.1./1.2 : Unclear allocation sequence / concealment	High<br><br>2.6/2.7 No ITT analysis, >10 % attrition	High<br><br>3.1 No ITT analysis, >10 % dropout	Low	Some concerns<br><br>5.1 No pre-specified analysis plan.	High
Montgomery, 2001 (15)	Low	Low	Low	Low	Low	Low
Montgomery, 1993 (16)	Low	Low	Low	Low	Low	Low
Nakatana, 2005 (17)	High<br><br>1.1 allocation not random	High<br><br>2.6/2.7 No ITT analysis, >10 % attrition	Some concerns<br><br>3.1 >10 % dropout	Low	Some concerns<br><br>5.1 No pre-specified analysis plan.	High
Stein, 2007 (18)	Low	Low	Low	Low	Low	Low
Tollefson, 1994 (19)	Low	Low	Low	Low	Some concerns<br><br>5.1 No pre-specified analysis plan.	Some concerns
Zohar, 1996 (20)	Some concerns	Low	Low	Low	Some concerns	Some concerns

	1.1./1.2 : Unclear allocation sequence / concealment				5.1 No pre-specified analysis plan.	

Meta-regression analysis

For studies included in our multiple metaregression, we used a multicollinearity test in order to avoid overfitting, whereby studies with a high correlation (r>0.8) would be excluded from the multiple meta-regression. As **table S2** shows, no studies were correlated to the degree of redundancy.

Table S2: multicollinearity testing

	**Publication Year**	**Trial arms**	**Sponsor status**	**High Risk of Bias**	**Clomipramine use**
Publication Year		-0.37	0.22	-0.22	-0.24
Trial arms	-0.37		-0.37	-0.53	-0.20
Sponsor status	0.22	-0.37		-0.63	-0.24
High Risk of Bias	-0.22	0.53	-0.63		0.40
Clomipramine Use	-0.24	-0.20	-0.19	0.40	

Using anova, we compared performance and correctness of fit of the different multiple meta-regression models. The multiple metaregression using clomipramine and high risk of bias performed significantly better than individual regression models (see table **S3**). Further increasing model complexity did not lead to a significantly better performance. Corrected Akaike's information criterion was lowest for the model using clomipramine and high risk of bias (see table **S4**). Using the parsimony principle, the metaregression with high risk of bias and clomipramine was preferred over more complex models. Notable, furthermore, is that even when using the most complex model including all metaregression variables, clomipramine remained a significant predictor (beta -0.39, 95%CI -0.70 to -0.076, p = 0.017).

Table S3: Model performance of metaregressions

Comparison of model performance	LRT	p-value
Clomipramine + High RoB vs. High RoB	6.9	0.009
Clomipramine + High RoB vs. Clomipramine	4.9	0.0276
Clomi + High RoB + sponsor status vs Clomipramine + High RoB	1.5	0.22
Clomipramine vs. High RoB vs Clomipramine + High RoB + publication year	2.8	0.10
Clomi + High RoB + sponsor status vs Clomi + High RoB + sponsor status + publication year	2.7	0.10
Clomi + High RoB + sponsor status vs. full model	4.2	0.12

LRT: likelihood ratio test statistic

Table S4: Akaike's information criterion of metaregressions

Variables in regression model	AICc
High risk of bias	26.1
Clomipramine use	24.1
High risk + clomipramine	21.8
High risk + clomipramine + publication year	21.8
High risk + clomipramine + sponsor status	23.2
Full model	23.6

AICc = Corrected Akaike's information criterion

Meta-regression of SSRI studies

As described in our main analysis, heterogeneity was low across SSRI studies (I squared = 16.0%, tau < 0.0001), and the test for heterogeneity was not significant (Q = 30, p = 0.23), suggesting the effect of SSRIs

compared to placebo to be consistent across studies. Meta-regressions for different SSRI's were not significant. Results persisted when considering a prediction interval (95% PI -0.55 to -0.39).

Figure S2 Forest plot for SSRI studies only

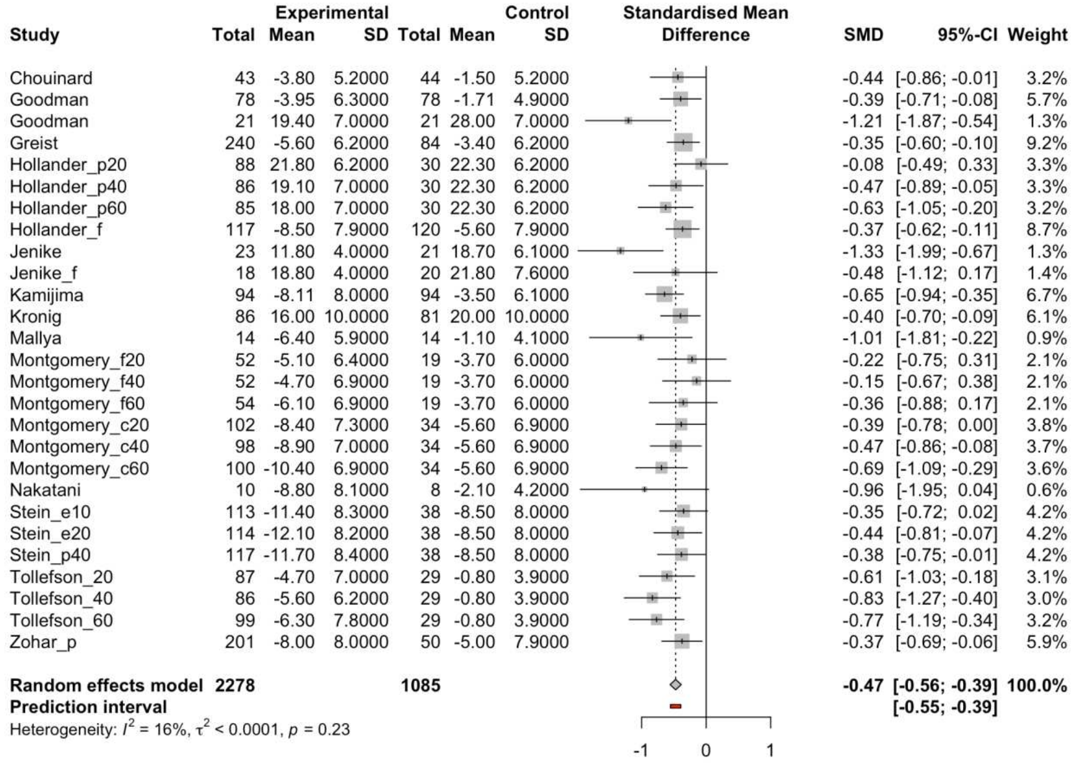

Publication bias

We used the robustbayesiancopas package in order to perform our Bayesian analysis of selection bias and used their proposed methods. We used multiple assumptions about distribution of the random effect (Student's T, Laplace, normal and slash distributions). Then, we extracted the Deviance Information Criterion (DIC) for each model to compare their goodness of fit. As slash distributions had the best fit (i.e. the lowest DIC), we used this distribution in further calculations., We then estimated the correlations parameter and fit a Bayesian model with and without correction for bias. We repeated our analysis multiple times using different seed settings which did not change the results. For SSRI studies only, using a Bayesian Copas selection model, we found a moderate effect of publication bias (D = 0.48) similarly to the full sample, with a decrease of 0.077 SMD, from – 0.48 (95% credible interval -0.57 to -0.40) to – 0.41 (95% credible interval -0.54 to -0.22).

Sensitivity analysis

After fully excluding all studies with a high risk of bias, clomipramine was still associated with a higher effect size (-0.38, p = 0.028, 95% CI =-0.72 to - 0.044), emphasizing the robustness of our finding that clomipramine has a higher efficacy than SSRI's when compared to placebo.

After combining intervention arms using different fixed doses, efficacy measures were comparable (SMD = -0.65, 95% CI -0.83 to -0.46). See **figure S3** for forest plot, including measures of heterogeneity. Furthermore, outcomes of meta-regression remained largely unchanged, except non-significance of the amount of intervention arms that were used. As our original analysis method increases the relative weight of studies with multiple intervention arms, the fact that in this analysis intervention arms are not significantly related to efficacy is an important addition to our original fidings. Please see **table S5** for single meta-regression results, and **table S6** for multiple metaregression including high risk of bias and clomipramine use. Furthermore, precision of estimates broadly decreased, with higher p-values, which is understandable considering the combination of doses decreases the degree of freedom for meta-regressions.

Figure S3 forest plot of studies with fixed doses combined in a single intervention arm.

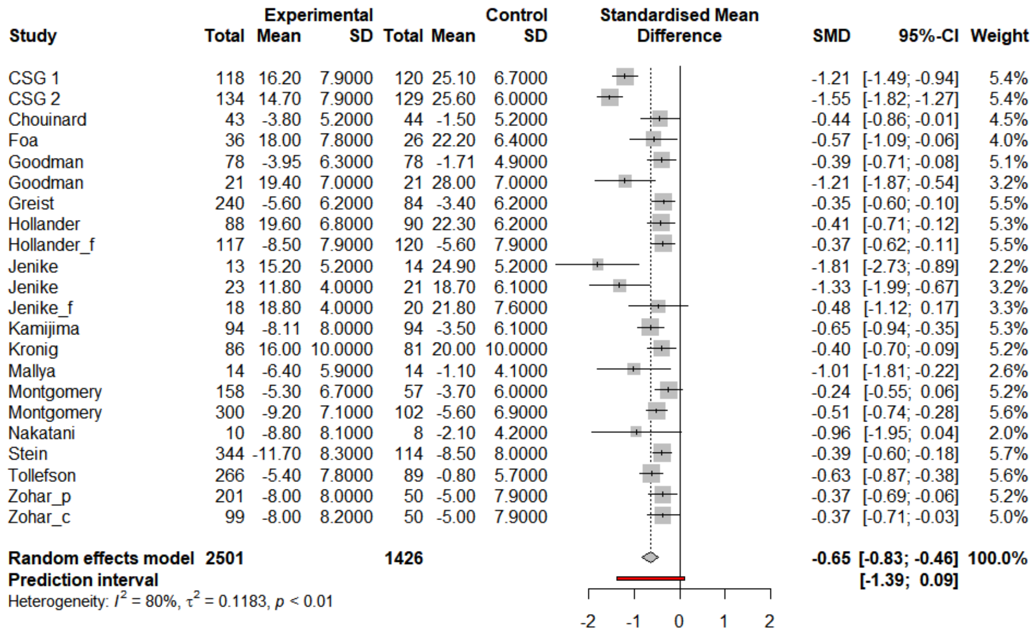

Table S5 single regression outcomes for combined fixed doses.

**Predictor**	**Beta-coefficient**	**P-value**	**95% CI Lower**	**95% CI Upper**
Categorical predictors				
High risk of bias	- 0.49	0.012	- 0.87	-0.12
Clomipramine use	-0.53	0.0073	-0.90	-0.16
Fully sponsored	0.44	0.041	0.020	0.87
Two-armed intervention trial	-0.33	0.060	-0.67	0.015
Use of placebo run-in	0.052	0.84	-0.47	0.58
Flexible dose	-0.25	0.17	-0.62	0.11
Continuous predictors				
Publication year	0.031	0.048	0.0030	0.062
Mean age	0.074	0.091	-0.013	0.16
Mean severity	-0.0038	0.94	-0.11	0.10
Duration of illness	-0.019	0.44	-0.072	0.034
Percentage male	0.0075	0.50	-0.015	0.030

Table S6 multiple regression outcomes for combined fixed doses

**Predictor**	**Beta-coefficient**	**p-Value**	**95% CI lower**	**95% CI upper**
High Risk of Bias	-0.34	0.069	-0.70	0.029
Clomipramine Use	-0.43	0.022	-0.79	-0.070

Table S7

**No of patients (studies)**	**Design**	**Limitations**	**Inconsistency**	**Indirectness**	**Imprecision**	**Publication Bias**
4102 (21)_	RCT’s	Serious limitations	Moderate inconsistency	Direct outcomes	Adequate precision	Suspicion of publication bias

GRADE assessment for quality of evidence, for outcome of YBOCS change at primary endpoint. All studies were placebo-controlled RCT’s. There were serious limitations due to most studies being at moderate risk of bias. Results were inconsistent, but less so for SSRI’s. Outcomes were direct, meaning population, intervention, or outcomes are comparable.

1. Chouinard G, Goodman W, Greist J, Jenike M, Rasmussen S, White K, et al. Results of a double-blind placebo controlled trial of a new serotonin uptake inhibitor, sertraline, in the treatment of obsessive-compulsive disorder. Psychopharmacol Bull. 1990;26(3):279-84.

2. Clomipramine in the treatment of patients with obsessive-compulsive disorder. The Clomipramine Collaborative Study Group. Arch Gen Psychiatry. 1991;48(8):730-8.

3. Foa EB, Liebowitz MR, Kozak MJ, Davies S, Campeas R, Franklin ME, et al. Randomized, placebo-controlled trial of exposure and ritual prevention, clomipramine, and their combination in the treatment of obsessive-compulsive disorder. Am J Psychiatry. 2005;162(1):151-61.

4. Goodman WK, Price LH, Rasmussen SA, Delgado PL, Heninger GR, Charney DS. Efficacy of fluvoxamine in obsessive-compulsive disorder. A double-blind comparison with placebo. Arch Gen Psychiatry. 1989;46(1):36-44.

5. Goodman WK, Kozak MJ, Liebowitz M, White KL. Treatment of obsessive-compulsive disorder with fluvoxamine: a multicentre, double-blind, placebo-controlled trial. Int Clin Psychopharmacol. 1996;11(1):21-9.

6. Greist J, Chouinard G, DuBoff E, Halaris A, Kim SW, Koran L, et al. Double-blind parallel comparison of three dosages of sertraline and placebo in outpatients with obsessive-compulsive disorder. Arch Gen Psychiatry. 1995;52(4):289-95.

7. Hollander E, Koran LM, Goodman WK, Greist JH, Ninan PT, Yang H, et al. A double-blind, placebo-controlled study of the efficacy and safety of controlled-release fluvoxamine in patients with obsessive-compulsive disorder. J Clin Psychiatry. 2003;64(6):640-7.

8. Hollander E, Allen A, Steiner M, Wheadon DE, Oakes R, Burnham DB. Acute and long-term treatment and prevention of relapse of obsessive-compulsive disorder with paroxetine. J Clin Psychiatry. 2003;64(9):1113-21.

9. Jenike MA, Baer L, Summergrad P, Weilburg JB, Holland A, Seymour R. Obsessive-compulsive disorder: a double-blind, placebo-controlled trial of clomipramine in 27 patients. Am J Psychiatry. 1989;146(10):1328-30.

10. Jenike MA, Hyman S, Baer L, Holland A, Minichiello WE, Buttolph L, et al. A controlled trial of fluvoxamine in obsessive-compulsive disorder: implications for a serotonergic theory. Am J Psychiatry. 1990;147(9):1209-15.

11. Jenike MA, Baer L, Minichiello WE, Rauch SL, Buttolph ML. Placebo-controlled trial of fluoxetine and phenelzine for obsessive-compulsive disorder. Am J Psychiatry. 1997;154(9):1261-4.

12. Kamijima K, Murasaki M, Asai M, Higuchi T, Nakajima T, Taga C, Matsunaga H. Paroxetine in the treatment of obsessive-compulsive disorder: randomized, double-blind, placebo-controlled study in Japanese patients. Psychiatry Clin Neurosci. 2004;58(4):427-33.

13. Kronig MH, Apter J, Asnis G, Bystritsky A, Curtis G, Ferguson J, et al. Placebo-controlled, multicenter study of sertraline treatment for obsessive-compulsive disorder. J Clin Psychopharmacol. 1999;19(2):172-6.

14. Mallya GK, White K, Waternaux C, Quay S. Short- and Long-Term Treatment of Obsessive-Compulsive Disorder with Fluvoxamine. Annals of Clinical Psychiatry. 1992;4(2):77-80.

15. Montgomery SA, Kasper S, Stein DJ, Bang Hedegaard K, Lemming OM. Citalopram 20 mg, 40 mg and 60 mg are all effective and well tolerated compared with placebo in obsessive-compulsive disorder. Int Clin Psychopharmacol. 2001;16(2):75-86.

16. Montgomery SA, McIntyre A, Osterheider M, Sarteschi P, Zitterl W, Zohar J, et al. A double-blind, placebo-controlled study of fluoxetine in patients with DSM-III-R obsessive-compulsive disorder. The Lilly European OCD Study Group. Eur Neuropsychopharmacol. 1993;3(2):143-52.

17. Nakatani E, Nakagawa A, Nakao T, Yoshizato C, Nabeyama M, Kudo A, et al. A randomized controlled trial of Japanese patients with obsessive-compulsive disorder--effectiveness of behavior therapy and fluvoxamine. Psychother Psychosom. 2005;74(5):269-76.

18. Stein DJ, Andersen EW, Tonnoir B, Fineberg N. Escitalopram in obsessive-compulsive disorder: a randomized, placebo-controlled, paroxetine-referenced, fixed-dose, 24-week study. Curr Med Res Opin. 2007;23(4):701-11.

19. Tollefson GD, Rampey AH, Jr., Potvin JH, Jenike MA, Rush AJ, kominguez RA, et al. A multicenter investigation of fixed-dose fluoxetine in the treatment of obsessive-compulsive disorder. Arch Gen Psychiatry. 1994;51(7):559-67.

20. Zohar J, Judge R. Paroxetine versus clomipramine in the treatment of obsessive-compulsive disorder. OCD Paroxetine Study Investigators. Br J Psychiatry. 1996;169(4):468-74.
